# Genetic variation and forensic characteristic analysis of 25 STRs of a novel fluorescence co-amplification system in Chinese Southern Shaanxi Han population

**DOI:** 10.18632/oncotarget.19317

**Published:** 2017-07-18

**Authors:** Yao-Shun Liu, Jian-Gang Chen, Ting Mei, Yu-Xin Guo, Hao-Tian Meng, Jian-Fei Li, Yuan-Yuan Wei, Xiao-Ye Jin, Bo-Feng Zhu, Li-Ping Zhang

**Affiliations:** ^1^ Department of Biochemistry and Molecular Biology, Basic Medicine College of Xinjiang Medical University, Urumqi, Xinjiang 830011, P. R. China; ^2^ Key Laboratory of Shaanxi Province for Craniofacial Precision Medicine Research, College of Stomatology, Xi’an Jiaotong University, Xi'an, Shaanxi 710004, P. R. China; ^3^ Clinical Research Center of Shaanxi Province for Dental and Maxillofacial Diseases, College of Stomatology, Xi’an Jiaotong University, Xi’an, Shaanxi 710004, P. R. China; ^4^ Department of Forensic Genetics, School of Forensic Medicine, Southern Medical University, Guangzhou, Guangdong 510515, P. R. China; ^5^ School of Marxism, Xi’an Jiaotong University, Xi’an, Shaanxi 710049, P.R. China; ^6^ Science and Technology Institute, Xinjiang Public Security Department, Urumqi, Xinjiang 830006, P.R. China

**Keywords:** genetic polymorphisms, Southern Shaanxi Han population, forensic characteristic analysis, autosomal STR, Y-STR

## Abstract

We analyzed the genetic polymorphisms of 15 autosomal and 10 Y-chromosomal STR loci in 214 individuals of Han population from Southern Shaanxi of China and studied the genetic relationships between Southern Shaanxi Han and other populations. We observed a total of 150 alleles at 15 autosomal STR loci with the corresponding allelic frequencies ranging from 0.0023 to 0.5210, and the combined power of discrimination and exclusion for the 15 autosomal STR loci were 0.99999999999999998866 and 0.999998491, respectively. For the 10 Y-STR loci, totally 100 different haplotypes were obtained, of which 94 were unique. The discriminatory capacity and haplotype diversity values of the 10 Y-STR loci were 0.9259 and 0.998269, respectively. The results demonstrated high genetic diversities of the 25 STR loci in the population for forensic applications. We constructed neighbor-joining tree and conducted principal component analysis based on 15 autosomal STR loci and conducted multidimensional scaling analysis and constructed neighbor-joining tree based on 10 Y-STR loci. The results of population genetic analyses based on both autosomal and Y-chromosome STRs indicated that the studied Southern Shaanxi Han population had relatively closer genetic relationship with Eastern Han population, and distant relationships with Croatian, Serbian and Moroccan populations.

## INTRODUCTION

Short tandem repeats (STRs) are widely distributed in human genome and play a significant role in forensic DNA analysis. Autosomal STRs have been the most common genetic markers in forensic applications and could be used to solve most of the personal identification and paternity testing cases [1, 2]. However, Y-chromosomal STRs (Y-STRs) would be more helpful in some special cases, such as mixed stain detection for sexual-assault cases, the paternal migration history tracing [3], and so on, because STRs on the non-recombining region of Y-chromosome do not participate in meiotic recombination and are unaltered when inherited from father to son [4–6]. At present, autosomal STRs or Y-STRs are often used alone in forensic applications. However, when autosomal STRs and Y-STRs were co-amplification in a single fluorescence multiplex system, they would be useful in both personal identification and paternity testing cases, for those Y-STRs could be helpful in the determination of gender and the reconstruction of paternal lineage [7, 8].

Southern Shaanxi region is the south part of Shaanxi province, covering the area from the south of Qinling Mountains to the north of Ba Mountain with the Han River flowing through from west to east; Southern Shaanxi consists of three cities: Hanzhong, Ankang and Shangluo, from west to east where mainly reside the Han population [9, 10]. To further understand the genetic background of Southern Shaanxi Han population and provide population genetic data for forensic identification, we firstly studied 15 autosomal STR loci and 10 Y-STR loci together in Chinese Han population from Southern Shaanxi region, calculated the forensic parameters, and collected previously published population data with overlapping loci of both autosomal STRs and Y-STRs, to discuss the genetic relationships between the studied population and other populations.

## RESULTS AND DISCUSSIONS

### The analysis of allelic frequencies and forensic parameters for the 15 autosomal STRs

According to the results of Hardy-Weinberg equilibrium (HWE) tests (presented in Table 1), all the autosomal STR loci showed no deviations from HWE (*p*>0.05). Tests of linkage disequilibrium (LD) were performed for all pairs of autosomal STR loci and the results were shown in Table 2. No LD was observed at a significance level of 0.0033 (α = 0.05/15) after Bonferroni correction, which indicated these autosomal STR loci were relatively independent. As summarized in Table 3, there were totally 150 alleles found at the 15 autosomal STR loci in the Southern Shaanxi Han population. As summarized in Table 1, the observed heterozygosity (Ho) in the studied population ranged from 0.6075 (TPOX locus) to 0.8411 (D8S1179 and D18S51 loci). The loci D18S51 and TPOX showed the highest and lowest expected heterozygosity (He), respectively. The power of discrimination (PD) and power of exclusion (PE) ranged from 0.7944 (TPOX locus) to 0.9623 (D2S1338 locus); and 0.2999 (TPOX locus) to 0.6774 (D8S1179 and D18S51 loci), respectively. The combined PD and PE were 0.99999999999999998866 and 0.999998491, respectively. All the 15 autosomal STR loci were found to be highly polymorphic in Southern Shaanxi Han population and the high value, which indicated their large potentiality for forensic individual identification.

**Table 1:** Forensic efficiency parameters of 15 autosomal STR loci in Southern Shaanxi Han (n = 214; 108 males and 106 females)

Loci	MP	PD	PIC	PE	TPI	Ho	He	*P*
D3S1358	0.1284	0.8716	0.6795	0.5058	1.9815	0.7477	0.7311	0.5469
D13S317	0.0762	0.9238	0.7753	0.6593	2.9722	0.8318	0.8079	0.3396
D7S820	0.0814	0.9186	0.7581	0.6060	2.5476	0.8037	0.7924	0.6354
D16S539	0.0742	0.9258	0.7677	0.6413	2.8158	0.8224	0.8020	0.4141
D19S433	0.0617	0.9383	0.7903	0.6235	2.6750	0.8131	0.8177	0.9201
TPOX	0.2056	0.7944	0.5485	0.2999	1.2738	0.6075	0.6167	0.8143
TH01	0.1652	0.8348	0.6147	0.3808	1.5070	0.6682	0.6629	0.8320
D2S1338	0.0377	0.9623	0.8432	0.6593	2.9722	0.8318	0.8630	0.2174
CSF1PO	0.1197	0.8803	0.6805	0.4593	1.7833	0.7196	0.7272	0.8481
vWA	0.0717	0.9283	0.7706	0.6060	2.5476	0.8037	0.8047	0.9718
D5S818	0.0812	0.9188	0.7535	0.5973	2.4884	0.7991	0.7897	0.6879
FGA	0.0413	0.9587	0.8361	0.6147	2.6098	0.8084	0.8559	0.0597
D8S1179	0.0426	0.9574	0.8321	0.6774	3.1471	0.8411	0.8541	0.6524
D21S11	0.0494	0.9506	0.8182	0.6683	3.0571	0.8364	0.8409	0.9216
D18S51	0.0401	0.9599	0.8468	0.6774	3.1471	0.8411	0.8658	0.3338

MP, matching probability; PD, power of discrimination; PIC, polymorphism information content; PE, probability of exclusion; TPI, typical paternity index; Ho, observed heterozygosity; He, expected heterozygosity; *P*, probability values of exact tests for Hardy–Weinberg equilibrium.

**Table 2:** *P*-value in pairwise linkage disequilibrium test at 15 autosomal STR loci in the Southern Shaanxi Han

Loci	D18S51	D21S11	D8S1179	FGA	D5S818	vWA	CSF1PO	D2S1338	TH01	TPOX	D19S433	D16S539	D7S820	D13S317
D21S11	0.6296													
D8S1179	0.5324	0.5499												
FGA	0.9829	0.9916	0.5286											
D5S818	0.5533	0.3886	0.1205	0.2850										
vWA	0.7924	0.5526	0.6802	0.6905	0.8789									
CSF1PO	0.4312	0.0657	0.5942	0.3433	0.1912	0.6657								
D2S1338	0.2196	0.0498	0.1329	0.5224	0.5909	0.2480	0.1339							
TH01	0.2324	0.8556	0.2354	0.0941	0.1334	0.1961	0.5523	0.7025						
TPOX	0.7221	0.8615	0.9330	0.5244	0.4134	0.0419	0.2683	0.0599	0.6112					
D19S433	0.8361	0.6447	0.4450	0.9006	0.6020	0.5437	0.5998	0.0914	0.5520	0.5389				
D16S539	0.1618	0.0346	0.3984	0.4926	0.0547	0.7402	0.7314	0.6914	0.8953	0.6968	0.9795			
D7S820	0.2090	0.7743	0.5845	0.6665	0.8183	0.2245	0.5539	0.7382	0.1664	0.7873	0.7797	0.9245		
D13S317	0.3965	0.8040	0.9324	0.8956	0.3898	0.9323	0.8857	0.7502	0.9283	0.7024	0.3950	0.2245	0.9659	
D3S1358	0.2112	0.3818	0.7652	0.9047	0.2617	0.6080	0.8065	0.2471	0.0870	0.1014	0.1553	0.0909	0.3529	0.8748

**Table 3:** Allele frequency distributions of 15 autosomal STR loci in Southern Shaanxi Han (n = 214; 108 males and 106 females)

Allele	D3S1358	Allele	FGA	Allele	D21S11	Allele	D19S433	Allele	D18S51	Allele	D2S1338	Allele	D5S818
14	0.0467	16	0.0023	16	0.0047	11	0.0023	10	0.0023	16	0.0187	7	0.0304
15	0.3341	18	0.0140	28	0.0607	12	0.0491	12	0.0467	17	0.0841	9	0.0701
16	0.3248	19	0.0607	28.2	0.0140	12.2	0.0070	13	0.2009	18	0.0771	10	0.2126
17	0.2220	20	0.0491	29	0.2336	13	0.2921	14	0.1939	19	0.1916	11	0.3014
18	0.0607	20.2	0.0023	30	0.2570	13.2	0.0304	15	0.1706	20	0.1192	12	0.2243
19	0.0093	21	0.0864	30.2	0.0164	14	0.2360	16	0.1145	21	0.0164	13	0.1472
20	0.0023	21.2	0.0047	30.3	0.0047	14.2	0.1215	17	0.0935	22	0.0514	14	0.0117
**Allele**	**D13S317**	22	0.1379	31	0.0981	15	0.0607	18	0.0374	23	0.2033	15	0.0023
8	0.2570	23	0.2593	31.2	0.0911	15.2	0.1472	19	0.0584	24	0.1706		
9	0.1449	23.2	0.0070	31.3	0.0023	16	0.0093	20	0.0444	25	0.0584		
10	0.1262	24	0.1776	32	0.0304	16.2	0.0397	21	0.0117	26	0.0070		
11	0.2453	24.2	0.0164	32.2	0.1308	17	0.0023	22	0.0117	27	0.0023		
12	0.1729	25	0.1168	33	0.0023	17.2	0.0023	23	0.0023	**Allele**	**TH01**		
13	0.0537	25.2	0.0047	33.2	0.0467	**Allele**	**D8S1179**	24	0.0070	6	0.1075		
**Allele**	**D7S820**	26	0.0491	34.2	0.0047	8	0.0023	25	0.0047	7	0.2570		
7	0.0047	27	0.0117	35.2	0.0023	9	0.0023	**Allele**	**CSF1PO**	8	0.0444		
8	0.1449	**Allele**	**D16S539**	**Allele**	**vWA**	10	0.1168	7	0.0070	9	0.5070		
9	0.0701	8	0.0093	14	0.2360	11	0.1005	9	0.0374	9.3	0.0444		
9.1	0.0047	9	0.2407	15	0.0164	12	0.0888	10	0.2547	10	0.0397		
10	0.1659	10	0.1449	16	0.1799	13	0.2196	11	0.1963	**Allele**	**TPOX**		
10.1	0.0047	11	0.2617	17	0.2523	14	0.1846	12	0.4065	8	0.5210		
11	0.3107	12	0.1963	18	0.1729	15	0.1519	13	0.0771	9	0.1192		
12	0.2430	13	0.1238	19	0.1308	16	0.1121	14	0.0117	10	0.0234		
13	0.0467	14	0.0210	20	0.0093	17	0.0187	15	0.0047	11	0.3154		
14	0.0047	15	0.0023	21	0.0023	18	0.0023	23	0.0047	12	0.0210		

### Interpopulation differentiations based on the 15 overlapping autosomal STRs

Fst statistics is one of the most widely used measures for genetic differentiation and plays a central role in genetic studies [11]. Reference populations including Hui [12,13], Uygur [14, 15], Eastern Han (from Zhejiang, China) [16, 17], Salar [18, 19], Miao [20, 21], Tibetan [22, 23], Yi [21, 24], Shandong Han [25, 26], Korean [27, 28], Bangladeshis [29, 30], Serbian [31, 32], Xibe [33, 34], Dong [21, 35], Maonan [21, 36], Moroccan [37, 38], Croatian [39, 40], Nepalese [41, 42], Jilin Han [43, 44] and Liaoning Han [45, 46] were used for population genetic analysis based on the same of autosomal STRs or Y-STR loci, respectively. The pairwise Fst and *p* values based on 15 STR loci between Southern Shaanxi Han population and other 19 populations were shown in Supplementary Table 1. The statistically significant differences (*p*<0.05/15=0.0033 after Bonferroni correction) were found between the Southern Shaanxi Han population and Serbian, Moroccan, Croatian, Miao, Shandong Han, Yi, Bangladeshis, Nepalese, Uygur and Dong populations at 11, 11, 10, 4, 3, 2, 2, 2, 1 and 1 loci, respectively. And then there was no significant difference obtained between Southern Shaanxi Han population and Eastern Han, Liaoning Han, Jilin Han, Hui, Salar, Tibetan, Xibe, Maonan and Korean populations, which indicated there were relatively close genetic distances among them.

### Principal component analysis based on the 15 overlapping autosomal STRs

**Figure 1:**
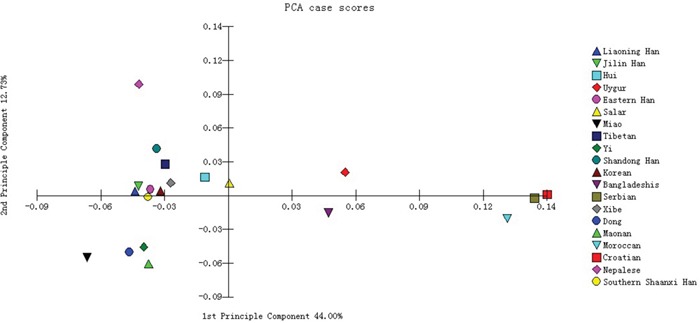
Principal component analysis based on the 15 overlapping autosomal STR loci of Southern Shaanxi Han population and 19 reference populations

The principal component analysis (PCA) was performed among the Southern Shaanxi Han population and other 19 populations using the allelic frequencies of the 15 overlapping autosomal STR loci. The PCA result was shown in Figure 1. The first and second components accounted for 44.00 and 12.73% of the total variance, respectively; and the cumulative contribution of them was 56.73%, which was over half of the total variance. According to Figure 1, Southern Shaanxi Han was located in left part, close to Eastern Han, Liaoning Han and Jilin Han population. Moroccan, Croatian and Serbian gathered in the right edge of the plot, which were relatively far away from Southern Shaanxi Han.

### Phylogenetic analysis based on the 15 overlapping autosomal STRs

**Figure 2:**
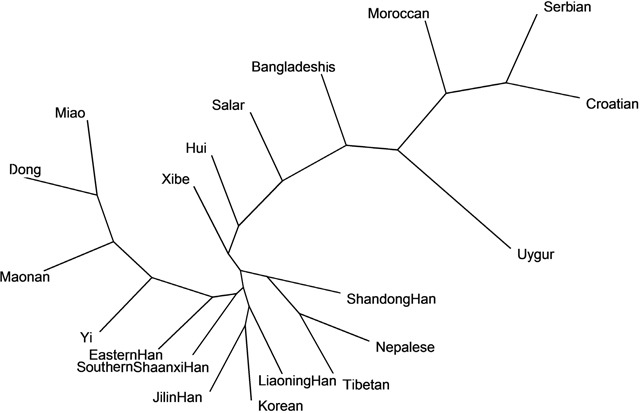
The neighbor-joining tree based on the 15 overlapping autosomal STR loci of Southern Shaanxi Han population and 19 reference populations

The neighbor-joining tree (NJ tree) of the Southern Shaanxi Han and other 19 populations based on allelic frequencies of 15 overlapping autosomal STR loci was shown in Figure 2. In the NJ tree, the Southern Shaanxi Han population was also observed to be close with the Eastern Han and Jilin Han population. However, Serbian, Croatian and Moroccan populations were located furthest away from Southern Shaanxi Han population. The phylogenetic result was consistent with the results of above-mentioned PCA.

### Allelic frequencies and haplotypic diversities of the 10 Y-STR loci

Allelic frequencies and Gene diversity (GD) values of the 10 Y-STR loci in 108 Southern Shaanxi Han male individuals were shown in Table 4, the allelic frequencies ranged from 0.0093 to 0.7407. GD values of all the loci were higher than 0.5 with the exceptions of DYS391 (0.4010) and DYS438 (0.3813) loci. The highest GD value was obtained at loci DYS385a, b with a value of 0.9983. The discriminatory capacity (DC) and haplotypic diversities (HD) values of the 10 Y-STR loci were 0.9259 and 0.9983, respectively. Haplotypic results of the 10 Y-STR loci were shown in Supplementary Table 2. One hundred different haplotypes were obtained, 94 of which were unique. We compared the 10 Y-STR haplotype data with the haplotype database in YHRD (http://www.yhrd.org) (Released March 01, 2017). Forty-one haplotypes detected in the Southern Shaanxi Han population were found with no matches in 131889 Haplotypes. Sixty-three haplotypes were found with matches in 31445 East Asian-Sino-Tibetan-Chinese and 17 haplotypes were found with matches in 3248 East Asian-Sino-Tibetan-Tibeto-Burman.

**Table 4:** Allele frequencies and Gene diversities (GD) for the 10 Y-STR loci in Southern Shaanxi Han (n = 108)

Allele	DYS635	DYS456	DYS458	DYS391	DYS392	DYS390	DYS393	DYS438	DYS385a,b			
8								0.0185	10,12	0.0093	13,17	0.0093
9				0.0185					10,17	0.0185	13,18	0.0926
10				0.7407	0.0093			0.7593	11,11	0.0370	13,19	0.0278
11				0.2315	0.1481			0.2130	11,12	0.0278	13,20	0.0093
12				0.0093	0.1481		0.5556	0.0093	11,13	0.0093	13,21	0.0278
13		0.0093	0.0093		0.3519		0.2593		11,16	0.0185	13,26	0.0093
14		0.2685	0.0185		0.2685		0.1111		11,17	0.0370	14,17	0.0185
15		0.4815	0.1296		0.0741		0.0741		11,18	0.0093	14,18	0.0185
15			0.0093						11,19	0.0370	14,19	0.0093
16		0.1574	0.1852						12,12	0.0370	14,21	0.0093
17		0.0741	0.2778						12,14	0.0093	14,22	0.0093
18		0.0093	0.2222						12,15	0.0093	15,17	0.0093
19	0.1389		0.1111						12,16	0.0741	15,19	0.0093
20	0.2778		0.0185			0.0093			12,17	0.0185	15,20	0.0093
21	0.2778		0.0093						12,19	0.0926	15,22	0.0093
22	0.2037		0.0093			0.0093			12,20	0.0741	18,19	0.0093
23	0.0556					0.5093			13,13	0.1204		
24	0.0370					0.2407			13,14	0.0370		
25	0.0093					0.2222			13,15	0.0093		
26						0.0093			13,16	0.0278		
GD	0.7876	0.6719	0.8165	0.4010	0.7617	0.6390	0.6120	0.3813		0.9983		

GD, gene diversity.

### Multidimensional scaling analysis based on the 10 Y- STRs

**Figure 3:**
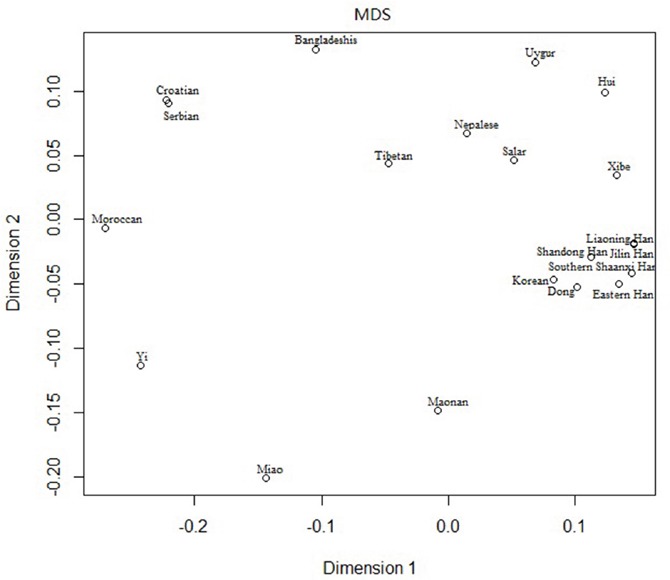
Multidimensional scaling analysis plot of the 20 populations based on the 10 overlapping Y-STR loci

The multidimensional scaling analysis (MDS) analysis was performed among the Southern Shaanxi Han population and 19 other populations based on *Rst* values at the 10 overlapping Y-STR loci in order to address population relationships, and the result was shown in Figure 3. According to the figure, Southern Shaanxi Han was located in right part, closest to Eastern Han population, Liaoning Han and Jilin Han. The result was similar to the above results of population genetic analyses based on the 15 overlapping autosomal STR loci.

### Phylogenetic analysis based on the 10 Y-STRs

**Figure 4:**
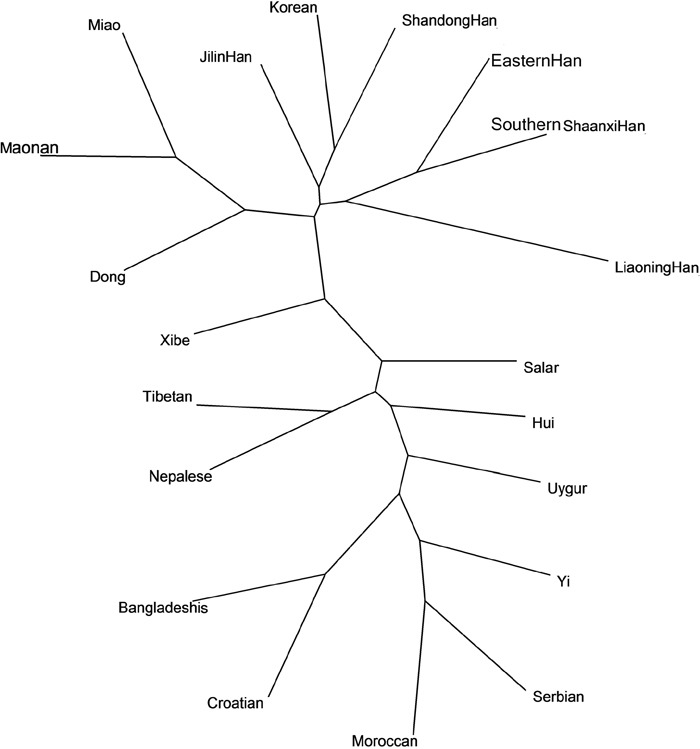
The neighbor-joining tree based on the 10 overlapping Y-STR loci of Southern Shaanxi Han population and 19 reference populations

As shown in Figure 4, the genetic relationships of the studied Southern Shaanxi Han and other 19 populations at the 10 overlapping Y-STRs were analyzed by the phylogenetic tree. In the NJ tree, the Southern Shaanxi Han population was also clustered close with the Eastern Han, Liaoning Han, Korean, Shandong Han and Xibe populations. Moreover, Croatian, Serbian, Moroccan and Yi populations were located furthest to Southern Shaanxi Han population. The phylogenetic result was similar to MDS result.

Southern Shaanxi Han speak Chinese, which belongs to Sino-Tibetan. Although settling in different areas, Southern Shaanxi Han and Eastern Han have a relatively close genetic relationship. However, for populations from other continents or races, such as Serbian, Moroccan, and Croatian, the genetic relationships with Southern Shaanxi Han would be quite distant because of their different origins and genetic structures. At present, there is little research on Southern Shaanxi Han population; more population genetic studies would help us get a better understanding of this population genetic backgroud in the future.

## MATERIALS AND METHODS

### Sample collection and DNA extraction

We collected 214 blood samples (108 males and 106 females) from unrelated healthy Han individuals living in the south of Shaanxi province (Southern Shaanxi), China. All the samples were collected according to the criteria for selection as follows: their ancestors within three generations should be unrelated individuals and members of Han ethnic group, with no migration. All volunteers without diseases signed informed consents. The present study followed the human rights and the ethical principle of Xinjiang Medical University and approved by institutional ethics committee, Xinjiang medical university, China. Genomic DNA was extracted using the Chelex-100 method as described by Walsh et al [47].

### Multiplex amplification and STR genotyping

The 25 STR loci (D3S1358, D13S317, D7S820, D16S539, D19S433, DYS635, DYS456, TPOX, TH01, D2S1338, CSF1PO, DYS385a,b, DYS458, DYS391, vWA, D5S818, FGA, DYS392, DYS390, D8S1179, D21S11, D18S51, DYS393 and DYS438) and Amelogenin locus were co-amplified in a new single multiplex reaction in a 25μL PCR reaction volume using the Expressmarker 16+10Y fluorescence amplification reagents (AGCU ScienTech Incorporation, Wuxi, Jiangsu, China), according to the manufacturer's instructions. DNA samples were amplified on a GeneAmp PCR System 9700 Thermal Cycler (Applied Biosystems, Foster City, CA, USA) following the manufacturer's instructions.

Electrophoresis was performed using an ABI 3130 Genetic Analyzer (Applied Biosystems, Foster City, CA, USA). Fragment sizing was supported using the AGCU Marker SIZ-500 (AGCU ScienTech Incorporation, Wuxi, Jiangsu, China) internal size standard and allelic ladder as basis for comparison. Alleles were identified using the GeneMapper® ID V3.2 (Applied Biosystems, Foster City, CA, USA). The 9948 cell-line (Promega, Madison, WI, USA) DNA was also genotyped using the reagent as control.

### Statistical analysis

Fifteen autosomal STRs were statistically analyzed as follows. We calculated the allelic frequencies and tested the Hardy-Weinberg equilibrium using the modified powerstat v1.2 spreadsheet [48]. The linkage disequilibrium of autosomal STRs was analyzed by Genepop v4.0.10 (http://genepop.curtin.edu.au/). The pairwise Fst and *p* values between the studied Han population and reference populations at 15 overlapping autosomal STR loci were estimated by the program ARLEQUIN v3.1 (http://cmpg.unibe.ch/software/arlequin3). Principal component analysis was performed with MVSP 3.1 (http://www.kovcomp.com) based on allelic frequencies of 15 overlapping autosomal STR loci, which was used to explore the extent of correlation genetic relationships. Neighbor-joining tree based on allelic frequencies of 15 overlapping autosomal STR loci were calculated using the Phylip-3.69 Software (http://evolution.gs.washington.edu/phylip.html). Based on the 10 overlapping Y-STR loci, another NJ tree was obtained by Phylip-3.69 Software (http://evolution.gs.washington.edu/phylip.html). Multidimensional scaling analysis of Y-STR based on *Rst* values was constructed by statistical software SPSS version 13.0 (SPSS Inc., Chicago, IL). Gene diversity and haplotype diversity were calculated using Nei's formula [49].

## CONCLUSION

In summary, the results demonstrated high genetic diversities of the 15 autosomal STR loci and 10 Y-STR loci in the Southern Shaanxi Han population and the studied polymorphic markers were suitable for forensic DNA cases, which could offer a new tool for forensic investigation. In this study, 15 autosome and 10 Y chromosome STR loci were amplified by the multi-color fluorescence technique in a single PCR reaction, and genotyping profile of both autosomal STR and Y chromosome STR loci could be obtained simultaneously. This made it possible for forensic geneticists to get the information of personal identification, gender determination and pedigree investigation in one step, which is a time, labor and cost saving strategy. The results of population differentiation, principal component analysis, multidimensional scaling analysis and phylogenic analysis indicated the Southern Shaanxi Han population had closer genetic relationship with the Eastern Han population and more distant relationships with populations from other races. The present data which combined autosomal STR loci and Y-STR loci will be useful for the enrichment of Chinese genetic information resources and provide valuable forensic data for forensic DNA cases.

## SUPPLEMENTARY MATERIALS FIGURES AND TABLES







## Abbreviations

HWE: Hardy-Weinberg equilibrium
LD: linkage disequilibrium
MP: matching probability
PD: power of discrimination
PIC: polymorphism information content
PE: probability of exclusion
TPI: typical paternity index
Ho: observed heterozygosity
He: expected heterozygosity
*p*: probability values of exact tests for Hardy–Weinberg equilibrium
NJ tree: neighbor-joining tree
PCA: principal component analysis
GD: Gene diversity
HD: haplotype diversity
DC: discriminatory capacity
MDS: Multidimensional scaling analysis.
