# Supplementary material for: Genetic variation and forensic characteristic analysis of 25 STRs of a novel fluorescence co-amplification system in Chinese Southern Shaanxi Han population
Source: Oncotarget. 2017 Jul 18;8(33):55443–52. doi: 10.18632/oncotarget.19317 (PMC5589671; doi:10.18632/oncotarget.19317)
Supplement: Supplementary file 1 [file oncotarget-08-55443-s001.pdf]

Genetic variation and forensic characteristic analysis of 25 STRs of a novel fluorescence co-amplification system in Chinese Southern Shaanxi Han population

SUPPLEMENTARY MATERIALS

Supplementary Table 1: Pairwise *Fst* and *p*-values between Southern Shaanxi Han and other groups at the 15 overlapping STR loci (n =214)

See Supplementary File 1

Supplementary Table 2: Y-STR haplotypes found in Southern Shaanxi Han at 10 loci level (n = 108)

See Supplementary File 2
