# Supplementary material for: Genetic variation and forensic characteristic analysis of 25 STRs of a novel fluorescence co-amplification system in Chinese Southern Shaanxi Han population
Source: Oncotarget. 2017 Jul 18;8(33):55443–52. doi: 10.18632/oncotarget.19317 (PMC5589671; doi:10.18632/oncotarget.19317)
Supplement: Supplementary file 2 [file oncotarget-08-55443-s002.docx]

Table S1. Pairwise *Fst* and *p*-values between Southern Shaanxi Han and other groups at the 15 overlapping STR loci (n =214).

| Populations | Index | D3S1358 | D13S317 | D7S820 | D16S539 | D19S433 | TPOX | TH01 | D2S1338 | CSF1PO | vWA | D5S818 | FGA | D8S1179 | D21S11 | D18S51 |
| --- | --- | --- | --- | --- | --- | --- | --- | --- | --- | --- | --- | --- | --- | --- | --- | --- |
| Liaoning Han | *Fst* | -0.0010 | -0.0016 | -0.0020 | 0.0003 | -0.0021 | -0.0023 | -0.0016 | -0.0001 | -0.0004 | -0.0006 | 0.0003 | -0.0009 | 0.0005 | -0.0003 | -0.0015 |
|  | *p* | 0.6396 | 0.8829 | 0.9460 | 0.3153 | 0.9910 | 0.9640 | 0.7838 | 0.4414 | 0.4775 | 0.6667 | 0.3063 | 0.7478 | 0.3153 | 0.5586 | 0.9099 |
| Jilin Han | *Fst* | -0.0006 | -0.0017 | -0.0017 | -0.0005 | -0.0020 | -0.0022 | -0.0016 | 0.0005 | 0.0000 | -0.0008 | -0.0003 | -0.0005 | 0.0005 | -0.0006 | -0.0009 |
|  | *p* | 0.5405 | 0.8739 | 0.9009 | 0.5225 | 0.9730 | 0.9640 | 0.8108 | 0.3153 | 0.3063 | 0.5856 | 0.4865 | 0.5496 | 0.3063 | 0.5496 | 0.6847 |
| Hui | *Fst* | -0.0053 | -0.0059 | -0.0070 | -0.0037 | -0.0016 | 0.0047 | -0.0034 | -0.0038 | 0.0008 | -0.0024 | 0.0005 | -0.0018 | -0.0033 | -0.0036 | 0.0022 |
|  | *p* | 0.8198 | 0.9279 | 0.9910 | 0.6937 | 0.5946 | 0.1982 | 0.6667 | 0.8378 | 0.4595 | 0.5676 | 0.2973 | 0.6396 | 0.7117 | 0.8739 | 0.1802 |
| Uygur | *Fst* | -0.0020 | 0.0027 | 0.0030 | 0.0002 | 0.0040 | 0.0063 | 0.0269 | 0.0058 | 0.0035 | 0.0085 | 0.0087 | 0.0027 | 0.0022 | -0.0020 | -0.0012 |
|  | *p* | 0.5135 | 0.3153 | 0.1892 | 0.3604 | 0.1532 | 0.1261 | **0.0000** | 0.0721 | 0.2162 | 0.0901 | 0.0721 | 0.1532 | 0.1892 | 0.5766 | 0.5135 |
| Eastern Han | *Fst* | -0.0039 | -0.0048 | -0.0025 | -0.0041 | -0.0059 | -0.0026 | -0.0063 | 0.0023 | 0.0014 | -0.0045 | -0.0059 | 0.0003 | -0.0035 | -0.0036 | 0.0009 |
|  | *p* | 0.7027 | 0.9009 | 0.5856 | 0.8198 | 0.9820 | 0.5496 | 0.9640 | 0.1802 | 0.3694 | 0.7748 | 0.9550 | 0.3784 | 0.8018 | 0.7478 | 0.3604 |
| Salar | *Fst* | -0.0067 | -0.0069 | -0.0039 | -0.0016 | -0.0006 | -0.0036 | 0.0007 | -0.0044 | 0.0014 | 0.0010 | -0.0063 | 0.0014 | -0.0021 | -0.0062 | -0.0034 |
|  | *p* | 0.9910 | 0.9910 | 0.7838 | 0.5315 | 0.4144 | 0.7207 | 0.2703 | 0.8919 | 0.3333 | 0.4324 | 0.9550 | 0.3243 | 0.6216 | 0.9910 | 0.7838 |
| Miao | *Fst* | -0.0014 | 0.0111 | 0.0096 | 0.0079 | 0.0060 | 0.0059 | -0.0012 | -0.0005 | 0.0002 | 0.0137 | -0.0024 | 0.0097 | 0.0092 | -0.0016 | 0.0025 |
|  | *p* | 0.7297 | **0.0000** | 0.0180 | 0.0180 | **0.0000** | 0.0811 | 0.5766 | 0.5856 | 0.4595 | **0.0000** | 0.9099 | **0.0000** | 0.0090 | 0.7478 | 0.1171 |
| Tibetan | *Fst* | -0.0019 | 0.0013 | -0.0015 | 0.0008 | -0.0003 | 0.0030 | -0.0008 | 0.0015 | -0.0010 | -0.0002 | 0.0009 | 0.0028 | 0.0034 | -0.0021 | 0.0035 |
|  | *p* | 0.8018 | 0.1802 | 0.7117 | 0.2703 | 0.4144 | 0.1171 | 0.5225 | 0.1351 | 0.6216 | 0.4865 | 0.2342 | 0.0270 | 0.0451 | 0.9550 | 0.0541 |
| Yi | *Fst* | -0.0033 | 0.0067 | 0.0352 | -0.0010 | -0.0026 | 0.0025 | -0.0003 | 0.0035 | 0.0077 | 0.0075 | -0.0023 | 0.0188 | 0.0062 | 0.0010 | -0.0017 |
|  | *p* | 0.8018 | 0.0541 | **0.0000** | 0.5496 | 0.7387 | 0.2342 | 0.4234 | 0.1532 | 0.0631 | 0.0451 | 0.6667 | **0.0000** | 0.0451 | 0.2793 | 0.7117 |
| Shandong Han | *Fst* | 0.0022 | -0.0004 | -0.0038 | 0.0219 | -0.0016 | -0.0032 | -0.0036 | 0.0008 | 0.1153 | 0.0028 | 0.0249 | -0.0033 | 0.0000 | -0.0022 | -0.0015 |
|  | *p* | 0.1351 | 0.4955 | 0.9460 | **0.0000** | 0.6757 | 0.8018 | 0.8919 | 0.2973 | **0.0000** | 0.1171 | **0.0000** | 0.9550 | 0.4505 | 0.7568 | 0.7027 |
| Korean | *Fst* | 0.0004 | -0.0021 | 0.0000 | 0.0001 | -0.0004 | -0.0015 | 0.0003 | 0.0011 | -0.0005 | 0.0010 | -0.0011 | -0.0003 | 0.0005 | 0.0046 | -0.0009 |
|  | *p* | 0.2432 | 0.9550 | 0.3874 | 0.4505 | 0.5225 | 0.6216 | 0.3153 | 0.1712 | 0.3694 | 0.2883 | 0.6126 | 0.3784 | 0.3333 | 0.0180 | 0.7027 |
| Bangladeshis | *Fst* | -0.0048 | 0.0012 | -0.0006 | 0.0016 | 0.0016 | 0.0189 | 0.0579 | 0.0015 | 0.0151 | 0.0008 | -0.0020 | 0.0010 | 0.0016 | 0.0017 | 0.0058 |
|  | *p* | 0.8649 | 0.2883 | 0.4234 | 0.2703 | 0.2432 | **0.0000** | **0.0000** | 0.2342 | 0.0090 | 0.3874 | 0.6306 | 0.3514 | 0.2883 | 0.2793 | 0.0721 |
| Serbian | *Fst* | 0.0090 | 0.0207 | 0.0199 | 0.0241 | 0.0219 | 0.0001 | 0.1210 | 0.0258 | 0.0137 | 0.0112 | 0.0269 | 0.0162 | 0.0143 | 0.0047 | 0.0043 |
|  | *p* | 0.0090 | **0.0000** | **0.0000** | **0.0000** | **0.0000** | 0.3784 | **0.0000** | **0.0000** | **0.0000** | **0.0000** | **0.0000** | **0.0000** | **0.0000** | 0.0270 | 0.0451 |
| Xibe | *Fst* | -0.0004 | -0.0037 | 0.0006 | -0.0031 | -0.0028 | -0.0031 | 0.0015 | -0.0009 | -0.0039 | -0.0014 | 0.0004 | -0.0035 | -0.0024 | -0.0015 | -0.0026 |
|  | *p* | 0.4595 | 0.9460 | 0.3333 | 0.8559 | 0.8649 | 0.8108 | 0.2523 | 0.5045 | 0.9279 | 0.6216 | 0.3333 | 0.9820 | 0.8108 | 0.6667 | 0.8829 |
| Dong | *Fst* | -0.0019 | 0.0033 | 0.0058 | -0.0007 | 0.0002 | -0.0016 | -0.0018 | -0.0007 | -0.0012 | 0.0436 | -0.0028 | 0.0050 | 0.0015 | -0.0016 | -0.0005 |
|  | *p* | 0.7658 | 0.0631 | 0.0451 | 0.5766 | 0.3333 | 0.6126 | 0.7478 | 0.5946 | 0.6126 | **0.0000** | 0.9820 | 0.0090 | 0.1802 | 0.7928 | 0.4865 |
| Maonan | *Fst* | -0.0058 | 0.0012 | 0.0034 | 0.0043 | -0.0061 | -0.0034 | 0.0013 | -0.0013 | -0.0055 | 0.0057 | -0.0032 | 0.0033 | 0.0062 | -0.0058 | 0.0005 |
|  | *p* | 0.8649 | 0.2973 | 0.1712 | 0.2252 | 0.9820 | 0.7297 | 0.3514 | 0.5405 | 0.9279 | 0.1261 | 0.6667 | 0.1261 | 0.0991 | 0.9820 | 0.3063 |
| Moroccan | *Fst* | 0.0036 | 0.0456 | 0.0154 | 0.0194 | 0.0185 | 0.0135 | 0.0557 | 0.0284 | 0.0309 | 0.0205 | 0.0328 | 0.0149 | 0.0007 | -0.0005 | 0.0126 |
|  | *p* | 0.0541 | **0.0000** | **0.0000** | **0.0000** | **0.0000** | **0.0000** | **0.0000** | **0.0000** | **0.0000** | 0.0090 | **0.0000** | **0.0000** | 0.3694 | 0.4685 | **0.0000** |
| Croatian | *Fst* | 0.0117 | 0.0148 | 0.0248 | 0.0246 | 0.0236 | 0.0002 | 0.1269 | 0.0270 | 0.0053 | 0.0171 | 0.0296 | 0.0182 | 0.0184 | 0.0062 | 0.0036 |
|  | *p* | 0.0090 | **0.0000** | **0.0000** | **0.0000** | **0.0000** | 0.3153 | **0.0000** | **0.0000** | 0.0991 | **0.0000** | **0.0000** | **0.0000** | **0.0000** | 0.0451 | 0.0451 |
| Nepalese | *Fst* | 0.0102 | 0.0253 | 0.0086 | -0.0033 | 0.0011 | -0.0051 | 0.0003 | 0.0033 | 0.0097 | -0.0017 | 0.0136 | 0.0020 | 0.0122 | -0.0023 | 0.0302 |
|  | *p* | 0.0811 | **0.0000** | 0.0360 | 0.7297 | 0.2883 | 0.8018 | 0.3784 | 0.1622 | 0.0451 | 0.5946 | 0.0270 | 0.2973 | 0.0180 | 0.7027 | **0.0000** |
