# Supplementary material for: Genetic variation and forensic characteristic analysis of 25 STRs of a novel fluorescence co-amplification system in Chinese Southern Shaanxi Han population
Source: Oncotarget. 2017 Jul 18;8(33):55443–52. doi: 10.18632/oncotarget.19317 (PMC5589671; doi:10.18632/oncotarget.19317)
Supplement: Supplementary file 3 [file oncotarget-08-55443-s003.docx]

Table S2. Y-STR haplotypes found in Southern Shaanxi Han at 10 loci level (n = 108).

| Haplotypes | Population | DYS635 | DYS456 | DYS385a,b | DYS458 | DYS391 | DYS392 | DYS390 | DYS393 | DYS438 | Number | Haplotype frequency |
| --- | --- | --- | --- | --- | --- | --- | --- | --- | --- | --- | --- | --- |
| H1 | Southern Shaanxi Han | 19 | 15 | 11,16 | 17 | 10 | 12 | 23 | 12 | 8 | 2 | 0.0185 |
| H2 | Southern Shaanxi Han | 19 | 15 | 11,17 | 19 | 9 | 12 | 23 | 12 | 10 | 1 | 0.0093 |
| H3 | Southern Shaanxi Han | 19 | 15 | 12,16 | 18 | 10 | 12 | 23 | 12 | 10 | 3 | 0.0278 |
| H4 | Southern Shaanxi Han | 19 | 15 | 13,13 | 16 | 10 | 15 | 23 | 14 | 10 | 1 | 0.0093 |
| H5 | Southern Shaanxi Han | 19 | 15 | 13,15 | 19 | 10 | 12 | 24 | 12 | 10 | 1 | 0.0093 |
| H6 | Southern Shaanxi Han | 19 | 16 | 12,12 | 19 | 10 | 12 | 23 | 12 | 10 | 1 | 0.0093 |
| H7 | Southern Shaanxi Han | 19 | 16 | 13,13 | 13 | 11 | 14 | 23 | 13 | 10 | 1 | 0.0093 |
| H8 | Southern Shaanxi Han | 19 | 17 | 13,13 | 15 | 11 | 14 | 23 | 13 | 10 | 2 | 0.0185 |
| H9 | Southern Shaanxi Han | 19 | 17 | 13,13 | 15 | 12 | 14 | 23 | 13 | 10 | 1 | 0.0093 |
| H10 | Southern Shaanxi Han | 19 | 17 | 13,13 | 16 | 11 | 14 | 23 | 13 | 10 | 2 | 0.0185 |
| H11 | Southern Shaanxi Han | 20 | 14 | 11,12 | 17 | 10 | 14 | 23 | 14 | 10 | 1 | 0.0093 |
| H12 | Southern Shaanxi Han | 20 | 14 | 12,12 | 18 | 10 | 14 | 24 | 13 | 10 | 1 | 0.0093 |
| H13 | Southern Shaanxi Han | 20 | 14 | 12,16 | 18 | 10 | 12 | 23 | 13 | 10 | 1 | 0.0093 |
| H14 | Southern Shaanxi Han | 20 | 14 | 12,19 | 17 | 10 | 13 | 25 | 12 | 10 | 1 | 0.0093 |
| H15 | Southern Shaanxi Han | 20 | 14 | 13,16 | 22 | 10 | 10 | 25 | 12 | 10 | 1 | 0.0093 |
| H16 | Southern Shaanxi Han | 20 | 14 | 13,21 | 17 | 10 | 14 | 23 | 12 | 11 | 1 | 0.0093 |
| H17 | Southern Shaanxi Han | 20 | 14 | 15,17 | 16 | 11 | 11 | 25 | 13 | 11 | 1 | 0.0093 |
| H18 | Southern Shaanxi Han | 20 | 15 | 12,15 | 18 | 10 | 12 | 23 | 13 | 10 | 1 | 0.0093 |
| H19 | Southern Shaanxi Han | 20 | 15 | 12,16 | 18 | 11 | 12 | 23 | 12 | 10 | 1 | 0.0093 |
| H20 | Southern Shaanxi Han | 20 | 15 | 13,14 | 17 | 10 | 13 | 23 | 13 | 10 | 1 | 0.0093 |
| H21 | Southern Shaanxi Han | 20 | 15 | 13,14 | 18 | 10 | 12 | 25 | 12 | 11 | 1 | 0.0093 |
| H22 | Southern Shaanxi Han | 20 | 15 | 13,18 | 18 | 10 | 14 | 23 | 12 | 11 | 1 | 0.0093 |
| H23 | Southern Shaanxi Han | 20 | 15 | 13,18 | 18 | 10 | 14 | 25 | 12 | 11 | 1 | 0.0093 |
| H24 | Southern Shaanxi Han | 20 | 15 | 13,19 | 17 | 10 | 14 | 23 | 12 | 11 | 1 | 0.0093 |
| H25 | Southern Shaanxi Han | 20 | 15 | 13,19 | 18 | 10 | 14 | 23 | 12 | 11 | 1 | 0.0093 |
| H26 | Southern Shaanxi Han | 20 | 15 | 13,19 | 18 | 10 | 15 | 24 | 12 | 11 | 1 | 0.0093 |
| H27 | Southern Shaanxi Han | 20 | 15 | 13,26 | 16 | 10 | 13 | 24 | 13 | 10 | 1 | 0.0093 |
| H28 | Southern Shaanxi Han | 20 | 15 | 14,21 | 19 | 10 | 15 | 25 | 13 | 11 | 1 | 0.0093 |
| H29 | Southern Shaanxi Han | 20 | 15 | 15,19 | 17 | 10 | 14 | 24 | 12 | 11 | 1 | 0.0093 |
| H30 | Southern Shaanxi Han | 20 | 16 | 13,13 | 16 | 10 | 14 | 23 | 13 | 10 | 1 | 0.0093 |
| H31 | Southern Shaanxi Han | 20 | 16 | 13,14 | 15 | 10 | 14 | 23 | 12 | 10 | 1 | 0.0093 |
| H32 | Southern Shaanxi Han | 20 | 16 | 13,16 | 16 | 10 | 15 | 23 | 13 | 11 | 1 | 0.0093 |
| H33 | Southern Shaanxi Han | 20 | 16 | 13,16 | 17 | 10 | 13 | 23 | 14 | 11 | 1 | 0.0093 |
| H34 | Southern Shaanxi Han | 20 | 16 | 13,18 | 17 | 10 | 14 | 23 | 12 | 11 | 1 | 0.0093 |
| H35 | Southern Shaanxi Han | 20 | 16 | 13,18 | 18 | 10 | 14 | 23 | 12 | 11 | 1 | 0.0093 |
| H36 | Southern Shaanxi Han | 20 | 16 | 13,20 | 18 | 10 | 14 | 24 | 12 | 11 | 1 | 0.0093 |
| H37 | Southern Shaanxi Han | 20 | 16 | 13,21 | 19 | 11 | 13 | 24 | 12 | 11 | 1 | 0.0093 |
| H38 | Southern Shaanxi Han | 20 | 17 | 12,16 | 19 | 10 | 12 | 23 | 12 | 11 | 1 | 0.0093 |
| H39 | Southern Shaanxi Han | 20 | 17 | 13,13 | 15 | 11 | 14 | 23 | 13 | 10 | 1 | 0.0093 |
| H40 | Southern Shaanxi Han | 20 | 18 | 12,16 | 16 | 11 | 12 | 23 | 12 | 10 | 1 | 0.0093 |
| H41 | Southern Shaanxi Han | 21 | 14 | 11,17 | 15 | 10 | 11 | 23 | 14 | 10 | 1 | 0.0093 |
| H42 | Southern Shaanxi Han | 21 | 14 | 11,19 | 16 | 10 | 11 | 23 | 13 | 10 | 2 | 0.0185 |
| H43 | Southern Shaanxi Han | 21 | 14 | 11,19 | 18 | 10 | 13 | 25 | 12 | 10 | 1 | 0.0093 |
| H44 | Southern Shaanxi Han | 21 | 14 | 12,19 | 16 | 10 | 13 | 23 | 12 | 10 | 1 | 0.0093 |
| H45 | Southern Shaanxi Han | 21 | 14 | 12,20 | 17 | 10 | 13 | 23 | 12 | 10 | 1 | 0.0093 |
| H46 | Southern Shaanxi Han | 21 | 14 | 12,20 | 17 | 11 | 13 | 25 | 12 | 10 | 1 | 0.0093 |
| H47 | Southern Shaanxi Han | 21 | 15 | 11,11 | 15 | 11 | 11 | 23 | 14 | 10 | 1 | 0.0093 |
| H48 | Southern Shaanxi Han | 21 | 15 | 11,13 | 15.1 | 10 | 14 | 23 | 14 | 10 | 1 | 0.0093 |
| H49 | Southern Shaanxi Han | 21 | 15 | 11,17 | 15 | 10 | 11 | 24 | 15 | 10 | 1 | 0.0093 |
| H50 | Southern Shaanxi Han | 21 | 15 | 11,17 | 16 | 10 | 11 | 20 | 15 | 10 | 1 | 0.0093 |
| H51 | Southern Shaanxi Han | 21 | 15 | 11,19 | 16 | 11 | 11 | 23 | 14 | 10 | 1 | 0.0093 |
| H52 | Southern Shaanxi Han | 21 | 15 | 12,12 | 17 | 11 | 15 | 23 | 13 | 10 | 1 | 0.0093 |
| H53 | Southern Shaanxi Han | 21 | 15 | 12,17 | 14 | 10 | 13 | 23 | 13 | 10 | 1 | 0.0093 |
| H54 | Southern Shaanxi Han | 21 | 15 | 12,17 | 15 | 10 | 13 | 24 | 15 | 10 | 1 | 0.0093 |
| H55 | Southern Shaanxi Han | 21 | 15 | 12,19 | 16 | 10 | 11 | 23 | 15 | 10 | 1 | 0.0093 |
| H56 | Southern Shaanxi Han | 21 | 15 | 12,19 | 19 | 11 | 13 | 25 | 13 | 10 | 1 | 0.0093 |
| H57 | Southern Shaanxi Han | 21 | 15 | 12,20 | 17 | 10 | 13 | 23 | 12 | 10 | 1 | 0.0093 |
| H58 | Southern Shaanxi Han | 21 | 15 | 13,18 | 17 | 11 | 14 | 24 | 12 | 11 | 1 | 0.0093 |
| H59 | Southern Shaanxi Han | 21 | 15 | 13,18 | 18 | 10 | 14 | 24 | 13 | 11 | 1 | 0.0093 |
| H60 | Southern Shaanxi Han | 21 | 15 | 14,18 | 17 | 10 | 11 | 23 | 12 | 10 | 1 | 0.0093 |
| H61 | Southern Shaanxi Han | 21 | 15 | 14,19 | 18 | 10 | 14 | 24 | 12 | 11 | 1 | 0.0093 |
| H62 | Southern Shaanxi Han | 21 | 15 | 14,22 | 19 | 10 | 15 | 25 | 12 | 10 | 1 | 0.0093 |
| H63 | Southern Shaanxi Han | 21 | 15 | 15,20 | 17 | 10 | 13 | 24 | 12 | 10 | 1 | 0.0093 |
| H64 | Southern Shaanxi Han | 21 | 16 | 11,11 | 15 | 10 | 11 | 23 | 15 | 10 | 1 | 0.0093 |
| H65 | Southern Shaanxi Han | 21 | 16 | 11,11 | 16 | 10 | 11 | 24 | 15 | 10 | 1 | 0.0093 |
| H66 | Southern Shaanxi Han | 21 | 16 | 13,13 | 16 | 10 | 15 | 23 | 12 | 10 | 1 | 0.0093 |
| H67 | Southern Shaanxi Han | 21 | 16 | 13,13 | 18 | 10 | 12 | 24 | 12 | 10 | 1 | 0.0093 |
| H68 | Southern Shaanxi Han | 21 | 16 | 13,14 | 16 | 10 | 15 | 23 | 13 | 10 | 1 | 0.0093 |
| H69 | Southern Shaanxi Han | 21 | 17 | 12,14 | 17 | 10 | 11 | 24 | 13 | 11 | 1 | 0.0093 |
| H70 | Southern Shaanxi Han | 22 | 13 | 10,17 | 17 | 10 | 13 | 24 | 12 | 10 | 1 | 0.0093 |
| H71 | Southern Shaanxi Han | 22 | 14 | 10,17 | 18 | 10 | 13 | 24 | 12 | 10 | 1 | 0.0093 |
| H72 | Southern Shaanxi Han | 22 | 14 | 12,19 | 18 | 10 | 13 | 25 | 12 | 10 | 3 | 0.0278 |
| H73 | Southern Shaanxi Han | 22 | 14 | 12,19 | 20 | 11 | 13 | 25 | 12 | 10 | 1 | 0.0093 |
| H74 | Southern Shaanxi Han | 22 | 14 | 12,20 | 17 | 10 | 13 | 24 | 12 | 10 | 1 | 0.0093 |
| H75 | Southern Shaanxi Han | 22 | 14 | 12,20 | 17 | 10 | 13 | 25 | 12 | 10 | 1 | 0.0093 |
| H76 | Southern Shaanxi Han | 22 | 14 | 12,20 | 18 | 10 | 13 | 25 | 12 | 10 | 1 | 0.0093 |
| H77 | Southern Shaanxi Han | 22 | 14 | 14,17 | 17 | 11 | 13 | 24 | 13 | 10 | 1 | 0.0093 |
| H78 | Southern Shaanxi Han | 22 | 15 | 10,12 | 16 | 11 | 14 | 23 | 13 | 10 | 1 | 0.0093 |
| H79 | Southern Shaanxi Han | 22 | 15 | 11,11 | 15 | 11 | 11 | 22 | 15 | 10 | 1 | 0.0093 |
| H80 | Southern Shaanxi Han | 22 | 15 | 11,12 | 15 | 10 | 11 | 23 | 15 | 10 | 1 | 0.0093 |
| H81 | Southern Shaanxi Han | 22 | 15 | 11,18 | 16 | 10 | 11 | 23 | 14 | 10 | 1 | 0.0093 |
| H82 | Southern Shaanxi Han | 22 | 15 | 12,12 | 17 | 11 | 13 | 25 | 12 | 10 | 1 | 0.0093 |
| H83 | Southern Shaanxi Han | 22 | 15 | 12,16 | 15 | 10 | 13 | 23 | 14 | 11 | 1 | 0.0093 |
| H84 | Southern Shaanxi Han | 22 | 15 | 12,20 | 19 | 11 | 13 | 23 | 12 | 10 | 1 | 0.0093 |
| H85 | Southern Shaanxi Han | 22 | 15 | 13,13 | 19 | 10 | 13 | 24 | 12 | 10 | 1 | 0.0093 |
| H86 | Southern Shaanxi Han | 22 | 15 | 13,18 | 18 | 11 | 13 | 25 | 14 | 10 | 1 | 0.0093 |
| H87 | Southern Shaanxi Han | 22 | 15 | 15,22 | 16 | 9 | 14 | 25 | 14 | 12 | 1 | 0.0093 |
| H88 | Southern Shaanxi Han | 22 | 15 | 18,19 | 21 | 10 | 12 | 26 | 12 | 10 | 1 | 0.0093 |
| H89 | Southern Shaanxi Han | 22 | 16 | 13,13 | 14 | 10 | 14 | 23 | 13 | 10 | 1 | 0.0093 |
| H90 | Southern Shaanxi Han | 23 | 14 | 12,19 | 18 | 10 | 13 | 25 | 12 | 10 | 1 | 0.0093 |
| H91 | Southern Shaanxi Han | 23 | 14 | 13,18 | 17 | 10 | 13 | 24 | 12 | 10 | 1 | 0.0093 |
| H92 | Southern Shaanxi Han | 23 | 14 | 14,18 | 17 | 10 | 13 | 25 | 12 | 10 | 1 | 0.0093 |
| H93 | Southern Shaanxi Han | 23 | 15 | 12,19 | 17 | 10 | 13 | 24 | 12 | 10 | 1 | 0.0093 |
| H94 | Southern Shaanxi Han | 23 | 15 | 13,18 | 17 | 11 | 13 | 24 | 14 | 10 | 1 | 0.0093 |
| H95 | Southern Shaanxi Han | 23 | 15 | 13,18 | 19 | 11 | 13 | 25 | 12 | 10 | 1 | 0.0093 |
| H96 | Southern Shaanxi Han | 24 | 14 | 13,17 | 17 | 10 | 13 | 25 | 12 | 10 | 1 | 0.0093 |
| H97 | Southern Shaanxi Han | 24 | 14 | 13,21 | 20 | 10 | 13 | 24 | 12 | 10 | 1 | 0.0093 |
| H98 | Southern Shaanxi Han | 24 | 14 | 14,17 | 17 | 10 | 14 | 25 | 12 | 10 | 1 | 0.0093 |
| H99 | Southern Shaanxi Han | 24 | 15 | 12,20 | 17 | 10 | 13 | 24 | 12 | 10 | 1 | 0.0093 |
| H100 | Southern Shaanxi Han | 25 | 16 | 11,12 | 15 | 10 | 14 | 23 | 13 | 11 | 1 | 0.0093 |
